# Multimodality Imaging of Gonococcal Pulmonary Valve Endocarditis

**DOI:** 10.1016/j.case.2026.04.002

**Published:** 2026-05-22

**Authors:** Michael Gomes, Denae Moore

**Affiliations:** a Department of Cardiology, Royal Perth Hospital, Perth, Western Australia, Australia; b Royal Darwin Hospital, Darwin, Northern Territory, Australia

**Keywords:** Infective endocarditis, Echocardiography, Transesophageal echocardiography, pulmonary valve, PET-CT

## Abstract

• PV IE can occur with gonococcal bacteremia.
• TTE is central for detecting PV vegetation and PR severity.
• FDG-PET supports diagnosis and defines extracardiac involvement.
• CT and PET can add value when TEE risk is increased.
• Individualized multimodality imaging is key in right-sided IE.

## Graphical abstract

**Figure:**
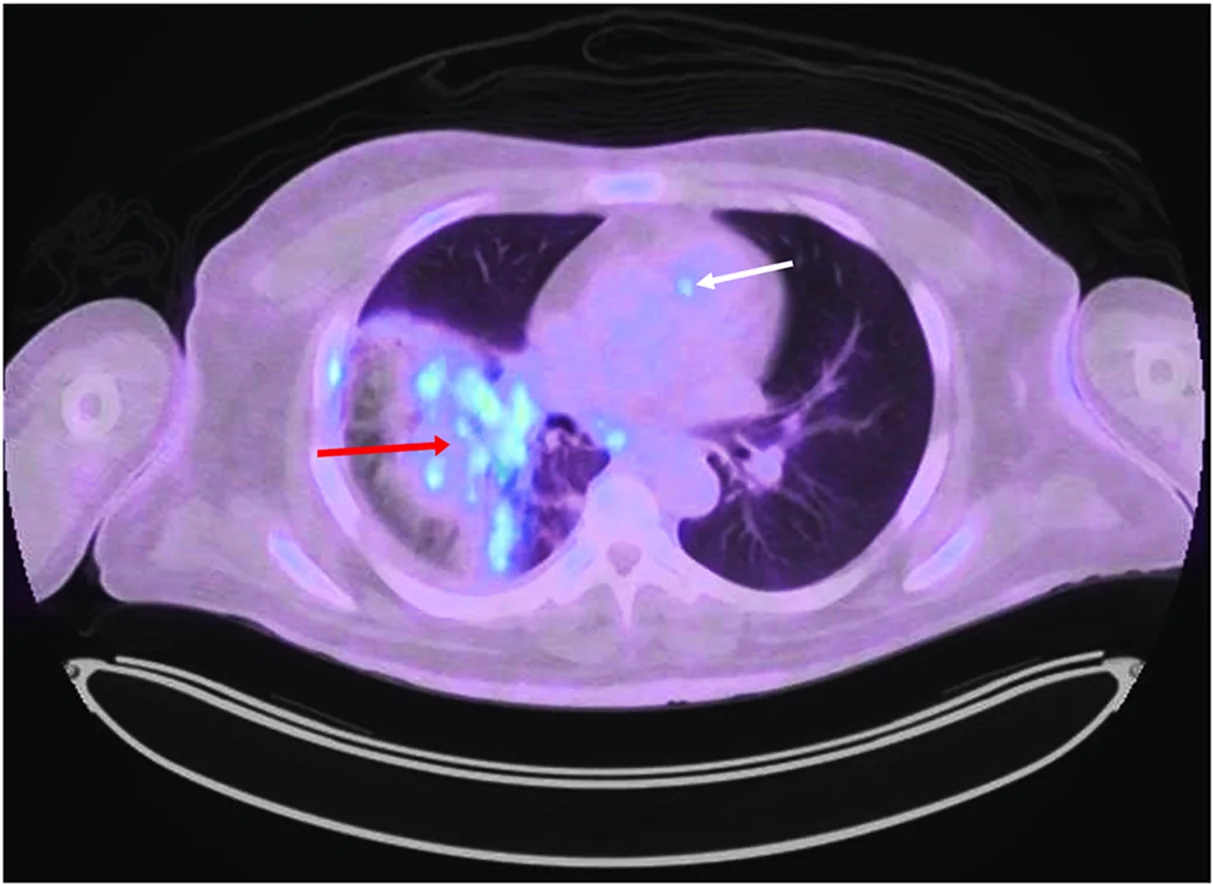

## Introduction

Pulmonary valve (PV) infective endocarditis (IE) is rare, accounting for less than 2% of IE cases, and is most commonly associated with intravenous drug use, congenital heart disease, or intracardiac devices.^1^^,^^2^ Right-sided disease presents unique diagnostic challenges, particularly when embolic complications predominate and standard imaging pathways are constrained. *Neisseria gonorrhoeae* endocarditis is also uncommon in the modern antibiotic era but may manifest with aggressive valvular destruction and systemic embolization.^3^

We describe a case of native PV endocarditis due to *N gonorrhoeae* complicated by septic pulmonary emboli and severe pulmonary regurgitation (PR). Transthoracic echocardiography (TTE) identified a mobile PV vegetation and quantified severe PR, establishing the diagnosis and hemodynamic severity. Cross-sectional imaging demonstrated splenic infarction and septic pulmonary emboli with pulmonary hemorrhage, while fluorodeoxyglucose (FDG) positron emission tomography (PET) confirmed metabolically active valvular infection and embolic involvement.^1^^,^^4^^,^^5^

Transesophageal echocardiography (TEE) was deferred due to esophageal varices and procedural risk. This case illustrates the diagnostic value of TTE in right-sided IE and highlights the complementary role of multimodality imaging in defining disease extent and guiding management.

## Case Presentation

A 37-year-old man presented with a 3 to 4 day history of fever, chills, night sweats, myalgia, malaise, and reduced oral intake. The patient also reported generalized weakness and urinary frequency. On examination, they were febrile and tachycardic, with a new systolic murmur best heard at the left upper sternal border, consistent with right ventricular (RV) outflow tract (RVOT) flow. There were no peripheral stigmata of IE.

Their medical history included type 2 diabetes mellitus and alcohol-related cirrhosis complicated by grade 1 esophageal varices. There was no history of intravenous drug use, prior cardiac surgery, prosthetic valves, intracardiac devices, or known congenital heart disease.

Laboratory evaluation demonstrated a marked systemic inflammatory response, with a C-reactive protein of 106 mg/L (reference <5 mg/L) on admission, peaking at 125.6 mg/L in the days following presentation. Leukocytosis (white cell count, 14.8 × 10^9^/L) and a neutrophilia (absolute neutrophil count, 12.6 × 10^9^/L) were present, and liver function tests were deranged in keeping with underlying cirrhosis.

Multiple sets of peripheral blood cultures obtained before antibiotic administration grew *N gonorrhoeae*. The organism was confirmed by nucleic acid amplification testing, with detection of *N gonorrhoeae* DNA on both blood culture confirmation testing and first-void urine polymerase chain reaction.

Serologic testing for atypical organisms was also performed. Bartonella serology returned positive, raising the possibility of a concomitant or alternative etiology of IE. In response, doxycycline was added to the antimicrobial regimen to provide coverage for potential Bartonella infection while further clinical correlation was undertaken.

**Figure 1:**
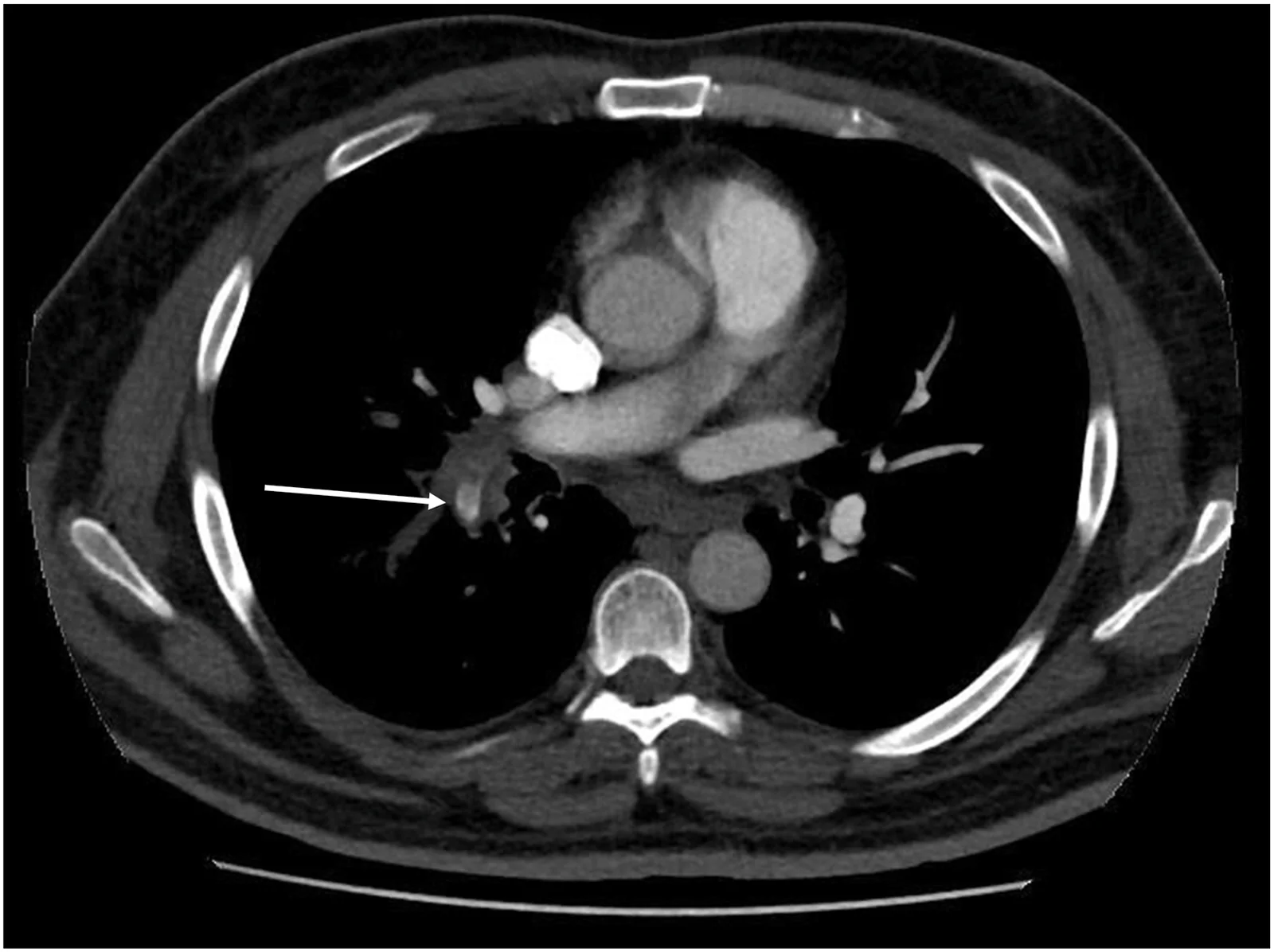
Computed tomography pulmonary angiogram (axial view) demonstrates segmental pulmonary arterial filling defects consistent with septic pulmonary emboli (*arrow*, filling defect in right pulmonary artery branch).

Given persistent tachycardia, evolving respiratory symptoms, and the development of hemoptysis, computed tomography (CT) pulmonary angiography was performed to evaluate for pulmonary embolism and pulmonary complications of infection. In the context of persistent bacteremia and concern for systemic embolic complications, CT imaging of the abdomen was performed to evaluate for intra-abdominal embolic or infective foci, demonstrating splenic infarction. Computed tomography pulmonary angiography revealed segmental and subsegmental pulmonary arterial thrombosis with associated consolidation and ground-glass changes consistent with pulmonary hemorrhage, in keeping with septic pulmonary emboli (Figure 1).

Following multidisciplinary discussion with the infectious diseases team, the overall clinical, microbiological, and imaging findings were considered most consistent with disseminated gonococcal infection complicated by PV endocarditis. The presence of persistent *N gonorrhoeae* bacteremia before treatment, concordant nucleic acid amplification testing confirmation, and the absence of other supportive clinical features of Bartonella endocarditis led to the conclusion that *N gonorrhoeae* was the causative pathogen.

Antimicrobial therapy was therefore continued with targeted intravenous ceftriaxone as the principal agent, with doxycycline discontinued once Bartonella infection was deemed unlikely. Repeat blood cultures obtained after initiation of targeted therapy demonstrated clearance of bacteremia, and inflammatory markers progressively declined in parallel with clinical improvement.

**Figure 2:**
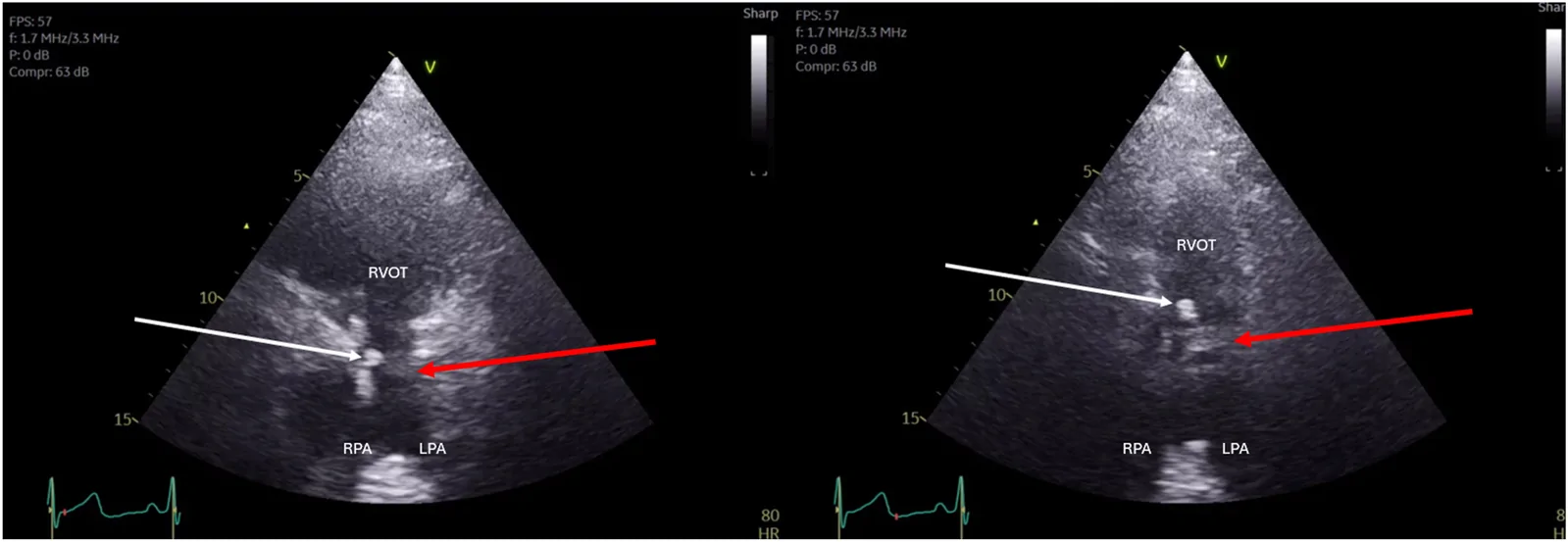
Two-dimensional TTE, basal parasternal short-axis systolic *(left)* and diastolic *(right)* views at the level of the RVOT, demonstrates a mobile echogenic mass *(white arrow)* attached to the PV *(red arrow)*, consistent with a vegetation. *LPA*, Left pulmonary artery; *RPA*, right pulmonary artery.

**Figure 3:**
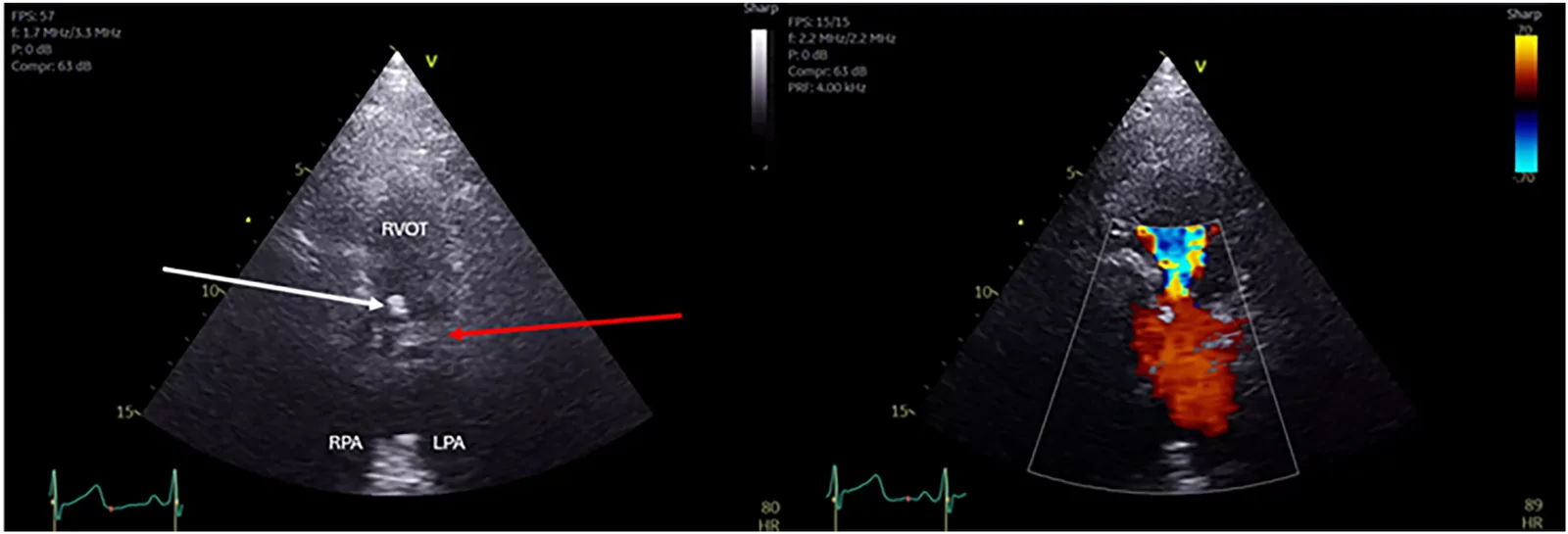
Two-dimensional TTE, basal parasternal short-axis diastolic view at the level of the RVOT without *(left)* and with *(right)* color-flow Doppler, demonstrates a mobile echogenic mass *(white arrow)* attached to the PV *(red arrow)*, consistent with a vegetation, and a broad regurgitant jet filling the RVOT, consistent with severe PR. *LPA*, Left pulmonary artery; *RPA*, right pulmonary artery.

**Figure 4:**
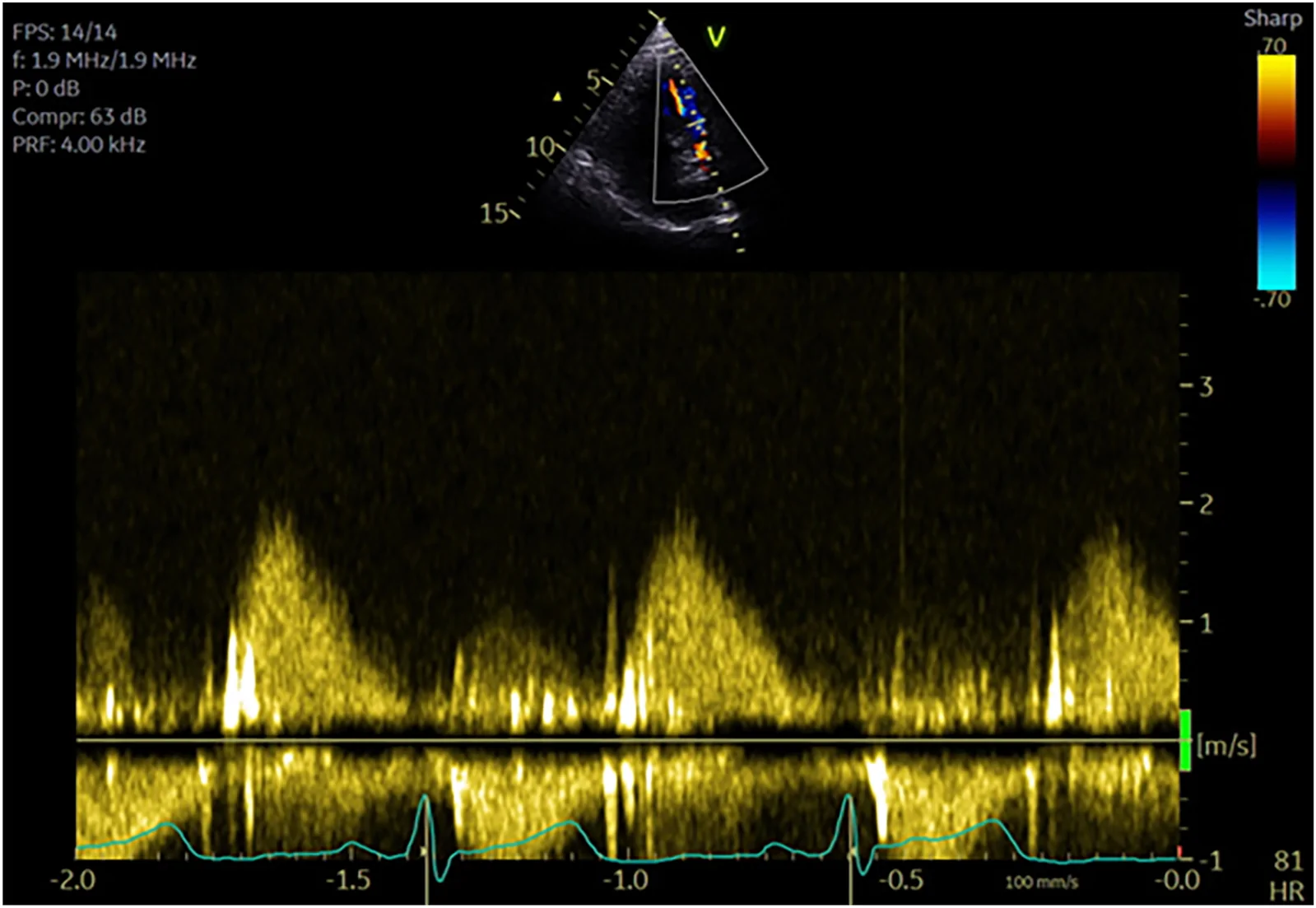
Continuous-wave spectral Doppler display with the cursor across the PV demonstrates a dense, diastolic spectral Doppler profile with rapid deceleration and early termination of flow, consistent with severe PR.

**Figure 5:**
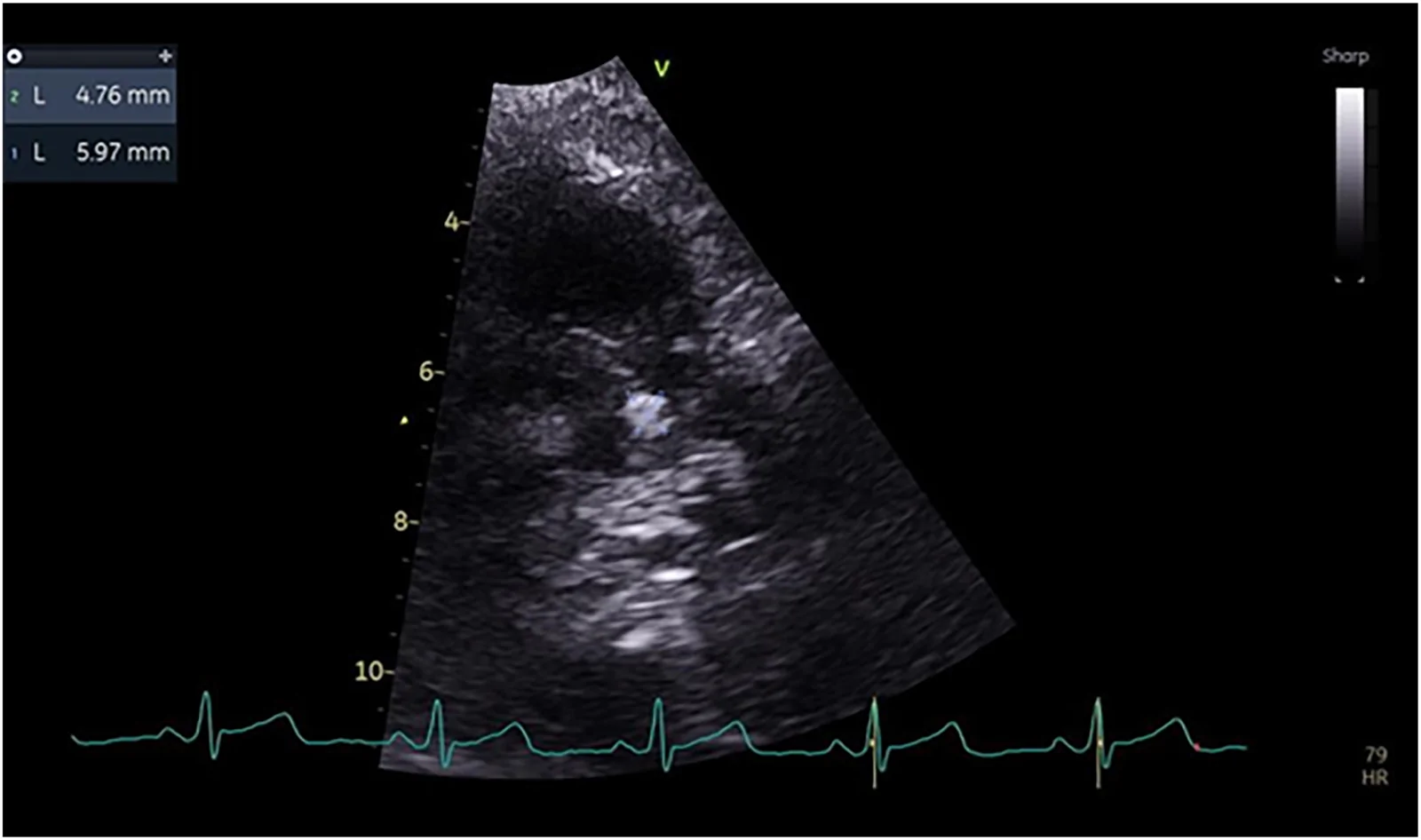
Two-dimensional TTE, basal parasternal short-axis diastolic view at the level of the RVOT obtained at 6 weeks’ follow- up, demonstrates a persistent echogenic mass (4.8 × 6.0 mm) attached to the PV *(red arrow)* consistent with a smaller vegetation.

Transthoracic echocardiography identified a mobile echodense mass on the PV with severe PR (Figure 2, Figure 3, Figure 4, Videos 1, 2). Repeat TTE confirmed multiple PV vegetations, the largest measuring approximately 0.8 × 0.7 cm, with RV volume loading. Color-flow Doppler demonstrated a broad regurgitant jet consistent with severe PR (Figure 3, Video 2). Continuous-wave Doppler showed a dense regurgitant signal with rapid deceleration (Figure 4). Follow-up TTE demonstrated persistent vegetations measuring up to 1.1 × 0.5 cm (Figure 5, Video 3).

**Figure 6:**
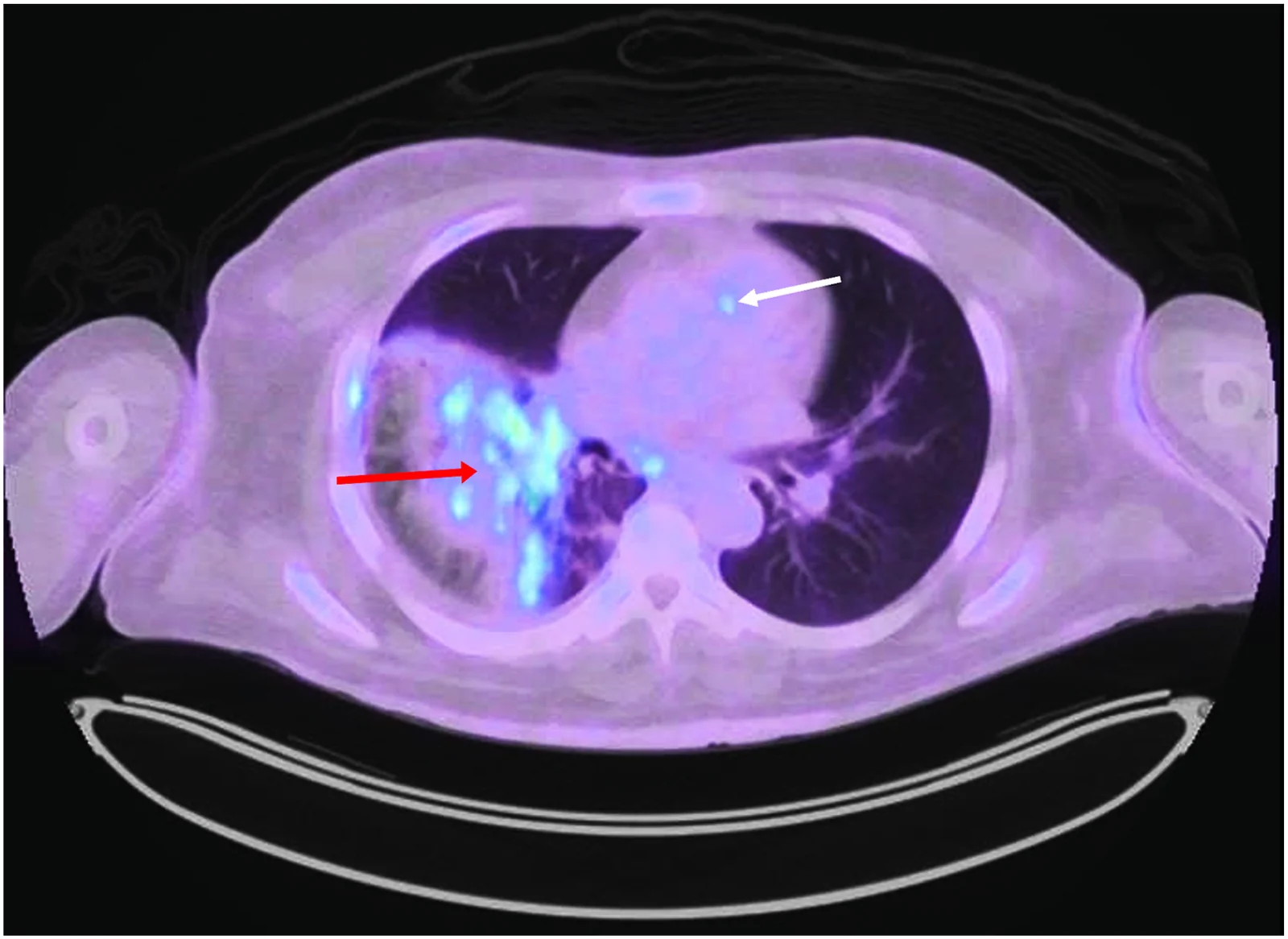
Fluorodeoxyglucose-PET/CT (axial fusion image) demonstrates focal hypermetabolic uptake at the PV *(white arrow)* and increased metabolic activity within the right lung consolidation *(red arrow)*, consistent with septic emboli.

Fluorodeoxyglucose-PET/CT was performed to assess for metabolically active infection and to define extracardiac disease burden in the context of disseminated infection. Fluorodeoxyglucose-PET/CT demonstrated focal hypermetabolic uptake at the PV corresponding to the echocardiographic vegetations and avid uptake in pulmonary consolidations, confirming active valvular infection and embolic inflammatory activity (Figure 6).

Transesophageal echocardiography was considered part of the diagnostic evaluation. The patient had known cirrhosis with previously documented grade 1 esophageal varices; however, the most recent endoscopic assessment was not available at the time of presentation, and the precise variceal location and burden were therefore uncertain.

A decision to defer TEE was made after multidisciplinary discussion and shared decision-making with the patient. Factors considered included the presence of portal hypertension, uncertainty regarding variceal burden and location in the absence of recent endoscopic assessment, and the anticipated incremental diagnostic yield. Although there was no history of recent gastrointestinal bleeding, TTE had already demonstrated a PV vegetation with severe regurgitation, and FDG-PET/CT showed concordant focal uptake at the PV and embolic pulmonary lesions. A noninvasive multimodality imaging strategy was therefore adopted in preference to TEE.

The patient completed 6 weeks of intravenous ceftriaxone under infectious diseases supervision. Management decisions, including the consideration and careful withholding of anticoagulation in the setting of pulmonary hemorrhage, were undertaken through a multidisciplinary endocarditis team comprising cardiology, infectious diseases, cardiothoracic surgery, and hepatology specialists. Clinical status progressively improved, with resolution of fever, clearance of bacteremia, and normalization of inflammatory markers.

Despite microbiological cure, severe PR persisted on follow-up TTE, with ongoing RV volume loading and residual mobile PV vegetation (Figure 5, Video 3). In the absence of hemodynamic instability, refractory infection, or progressive right heart failure, a conservative strategy was adopted. The patient was discharged with close cardiology follow-up, including serial TTE to monitor RV size and function and the severity of PR. Cardiovascular magnetic resonance imaging was planned to provide quantitative assessment of RV volumes and regurgitant fraction.

Ongoing surveillance was recommended given the risk of progressive RV dilation and dysfunction associated with severe PR. Consideration of PV intervention, including surgical valve replacement or transcatheter PV implantation, will be guided by the development of symptoms, evidence of RV enlargement or dysfunction on imaging, or worsening hemodynamic burden.

## Discussion

Pulmonary valve IE is rare, representing <2% of cases, and is typically associated with intravenous drug use, congenital heart disease, or devices.^2^^,^^3^^,^^6^ Gonococcal endocarditis is also uncommon but increasingly reported and may cause destructive valvular infection with embolic complications.^7^, ^8^, ^9^

The presence of splenic infarction in this case is atypical for isolated right-sided IE and raises the consideration of alternative mechanisms. Potential explanations include paradoxical embolization through an undetected intracardiac shunt, hematogenous dissemination in the context of severe bacteremia, or embolic phenomena related to disseminated gonococcal infection. In the absence of clinical or imaging evidence of an intracardiac shunt, hematogenous spread remains the most likely mechanism.

Pulmonary valve endocarditis can be challenging to detect on initial imaging. Guidelines emphasize that when clinical suspicion remains high, repeat echocardiography and complementary imaging modalities are essential.^1^^,^^5^^,^^10^

Although TEE is commonly recommended when transthoracic imaging is nondiagnostic or when clinical suspicion for IE remains high, current European Society of Cardiology and American Heart Association guidelines emphasize that imaging strategies should be individualized according to clinical context, image quality, and patient risk.^1^^,^^10^ The PV is anatomically anterior and may not be optimally visualized on TEE, whereas TTE often provides adequate assessment of right-sided structures.

According to American Society of Echocardiography guidelines, esophageal varices represent a relative rather than absolute contraindication to TEE, and the procedure may be performed safely in selected patients following careful risk assessment. Several observational studies and systematic reviews, including a recent meta-analysis, have demonstrated a low incidence of clinically significant gastrointestinal bleeding in patients with esophageal varices undergoing TEE.^11^, ^12^, ^13^

Although 18F-FDG PET/CT is well established in prosthetic valve endocarditis, its diagnostic performance in native valve IE is more limited, with reduced sensitivity related to smaller vegetations and cardiac motion.^14^^,^^15^ Current guidelines therefore do not recommend PET as a first-line imaging modality in native valve disease but support its use in selected cases, particularly when there is suspicion of extracardiac complications or when additional diagnostic confirmation is required.^1^

In this case, PET/CT was performed in the context of systemic embolic phenomena and provided important incremental value. The presence of focal hypermetabolic uptake at the PV, together with metabolically active pulmonary lesions, supported the diagnosis of active infection and septic embolization. Rather than serving as a primary diagnostic modality, PET functioned as a complementary tool to TTE, increasing diagnostic confidence and helping define the full extent of disease.

Septic pulmonary emboli complicated by pulmonary hemorrhage create complex anticoagulation decisions that require careful, individualized risk-benefit assessment.^1^^,^^3^^,^^10^ While standard anticoagulation is generally indicated for acute pulmonary embolism, the presence of infection-related vascular injury and active pulmonary hemorrhage substantially increases bleeding risk. Current IE guidelines do not provide specific recommendations for anticoagulation in septic pulmonary embolism, and management must therefore be individualized through multidisciplinary discussion, balancing thrombotic burden against hemorrhagic risk.

Right-sided endocarditis surgical decisions differ from left-sided disease and benefit from structured endocarditis team management.^1^^,^^5^ Pulmonary valve involvement presents distinct diagnostic and management considerations compared with more common left-sided disease. Because PV endocarditis is rare, it may not be immediately suspected, particularly in patients without intravenous drug use, prosthetic material, or known structural heart disease. Concurrently, disseminated gonococcal infection remains an uncommon but important cause of invasive endovascular infection, and cardiac involvement may occur even when initial presentation is nonspecific.

In this setting, multimodality imaging has incremental value beyond single-technique assessment. Fluorodeoxyglucose-PET may provide supportive evidence of active infection, demonstrate extracardiac embolic burden, and increase diagnostic confidence when used as an adjunct to echocardiography. Within a broader clinical and multidisciplinary assessment, this integrated imaging approach can support a noninvasive strategy in selected patients with elevated procedural risk. A structured endocarditis team approach is particularly important in these uncommon presentations to integrate microbiology, imaging, and surgical risk when planning therapy and follow-up.

## Conclusion

Pulmonary valve IE due to *N gonorrhoeae* is an uncommon presentation that may occur on native valves in patients without traditional right-sided risk factors. Recognition requires a high index of suspicion when bacteremia is accompanied by right-sided murmurs or pulmonary embolic phenomena.^1^, ^2^, ^3^^,^^8^, ^9^, ^10^

This case highlights the importance of individualized imaging strategies in right-sided IE. Transthoracic echocardiography can establish diagnosis and define hemodynamic severity when image quality is adequate, while adjunctive imaging modalities may provide additional diagnostic confidence and delineate extracardiac disease burden in selected cases.^1^^,^^4^^,^^14^^,^^15^

Careful integration of clinical, microbiological, and imaging findings, supported by multidisciplinary endocarditis team management, is essential to guide therapy, surveillance, and timing of potential intervention in these uncommon presentations.^1^^,^^5^^,^^10^

## Consent Statement

Complete written informed consent was obtained from the patient (or appropriate parent, guardian, or power of attorney) for the publication of this study and accompanying images.

## Ethics Statement

The authors declare that the work described has been carried out in accordance with The Code of Ethics of the World Medical Association (Declaration of Helsinki) for experiments involving humans

## Disclosure Statement

The authors reported no actual or potential conflicts of interest relative to this document.

## Supplementary Data

Video 1 Two-dimensional TTE, basal parasternal short-axis view at the level of the RVOT, demonstrates a mobile echogenic mass attached to the PV consistent with a vegetation.

Video 2 Two-dimensional TTE, basal parasternal short-axis view at the level of the RVOT with color-flow Doppler, demonstrates a mobile echogenic mass attached to the PV consistent with a vegetation, and a broad, regurgitant jet filling the RVOT consistent with severe PR.

Video 3 Two-dimensional TTE, basal parasternal short-axis diastolic view at the level of the RVOT obtained at 6 weeks’ follow-up, demonstrates a persistent echogenic mass (4.8 × 6.0 mm) attached to the PV consistent with a smaller vegetation.
